# Supplementary material for: Shear stress activates ADAM10 sheddase to regulate Notch1 via the Piezo1 force sensor in endothelial cells
Source: eLife. 2020 Jun 2;9:e50684. doi: 10.7554/eLife.50684 (PMC7295575; doi:10.7554/eLife.50684)
Supplement: Supplementary file 1. [file elife-50684-supp1.docx]

**Supplementary File 1. PCR primer sequences.**

| **Species** | **Gene** | **Forward** | **Reverse** |
| --- | --- | --- | --- |
| **Homo Sapiens** | *GAPDH* | GCCTCAAGATCATCAGCAAT | GGACTGTGGTCATGAGTCCT |
|  | *DLL4* | ACAACTTGTCGGACTTCCAG | CAGCTCCTTCTTCTGGTTTG |
|  | *JAG1* | GCGTTGCCCACTTTGAGTAT | GCCTTCCATGCAAGTTTTGT |
|  | *HES1*  *HES2* | CCAAAGACAGCATCTGAGCA  CGCATCAACCAGAGCCTG | GCCGCGAGCTATCTTTCTT  GAGCAGTTGGAGTTCTCCCG |
|  | *HEY1* | GCGTGGGAAAGGATGGTTGAG | TCCGCTCTCGGCTGCTTG |
|  | *HEY2* | GAAGATGCTTCAGGCAACAG | GCAACTTCTGTTAGGCACTCTC |
|  | *PIEZO1* | CGTCTTCGTGGAGCAGATG | GCCCTTGACGGTGCATAC |
|  | *ADAM10* | TTGCCTCCTCCTAAACCACTTCCA | AGGCAGTAGGAAGAACCAAGGCAA |
| **Mus Musculus** | *Actb* | CCAGATCTTCTCCATGTCGT | CCAGATCTTCTCCATGTCGT |
|  | *Dll4*  *Jag1* | ACTTCGTCTGCAACTGTCCT  CCTCTGCTGAGCTCTGTCTT | CAGCACCAGCAGTACCACTA  ATTGTTGGTGGTGTTGTCCT |
|  | *Hes1* | CCTCTGAGCACAGAAAGTCA | GCCGGGAGCTATCTTTCTTA |
|  | *Hey1*  *HeyL*  *Hes2*  *Hes3* | GTACCCAGTGCCTTTGAGAA  GTCCTTCCTGCATAGCTGTC  AGCTGAAGGGTCTCGTATTG  ACCTCTGTTCTCAACCCTTG | TTTCAGGTGATCCACAGTCA  GGTAAGGGGGAGAAGAGATG  GTAGGAAGCGCACAGTCATT  ACAACATCGGTGGAAGACTC |
|  | *Efnb2* | GGCCTGGTACTATACCCACA | ATTGTGCATCTGTCTGCTTG |
|  | *Piezo1* | TGAGCCCTTCCCCAACAATAC | CTGCAGGTGGTTCTGGATATAG |
|  | *Tek* | AAGCAACCCAGCCTTTTCTC | TGAGCATTCTCCTTTGGAC |

**Supplement Figure 9**

**Uncropped western blots for the supplement figures 1, 2 and 3.**
